# Cardiovascular Diseases and Schizophrenia in India: Evidence, Gaps, and Way Forward

**DOI:** 10.3389/fpsyt.2021.639295

**Published:** 2021-06-24

**Authors:** Ramachandran Padmavati, Suvarna Jyothi Kantipudi, Suhavana Balasubramanian, Vijaya Raghavan

**Affiliations:** ^1^Schizophrenia Research Foundation, Chennai, India; ^2^Department of Psychiatry, Sri Ramachandra Institute of Higher Education and Research, Chennai, India

**Keywords:** cardiovascular diseases, risk factors, interventions, India, schizophrenia

## Abstract

**Background:** The importance of physical health among persons with schizophrenia is well-established. Studies from developed and developing countries indicated a strong association between cardiovascular diseases and schizophrenia, while evidence from India is scattered and in its infancy. Hence, the aims of the study were to collate available studies from India on cardiovascular diseases among persons with schizophrenia, identify knowledge gaps and challenges, and discuss recommendations to improve clinical care and research on cardiovascular diseases among persons with schizophrenia in India.

**Materials and methods:** A comprehensive literature review of Indian studies on cardiovascular diseases and schizophrenia was conducted to collate and synthesise available knowledge.

**Results:** Several risk factors for cardiovascular disease predominated among persons with schizophrenia. Metabolic syndrome and obesity were the key factors that were reported. Knowledge gaps were identified with respect to the prevalence of cardiovascular diseases among persons with schizophrenia. Sparse research in interventions to prevent and reduce the impact of cardiovascular diseases among persons with schizophrenia was noted.

**Conclusion:** Targeted efforts are needed at the clinic, community, and policy levels to understand the impact of cardiovascular diseases among persons with schizophrenia. Robust and feasible interventions targeting cardiovascular diseases and its varied risk factors in persons with schizophrenia, that can be implemented in tertiary mental health services, need to be developed and tested.

## Introduction

Cardiovascular diseases (CVD) are the leading cause of morbidity and mortality in India (1). Serious mental disorders (SMD), comprising of schizophrenia spectrum disorders and bipolar disorders, also contribute as major causes of morbidity worldwide (2) and in India (3). Among the serious mental disorders, CVD morbidity and mortality is more pronounced among schizophrenia spectrum disorders. In the National Mental Health Survey in 2016, the prevalence of schizophrenia spectrum disorders was found to be 0.8% in India (4). This translates to a huge number of people being affected by schizophrenia spectrum disorders in India.

While global literature has shown a strong association between cardiovascular diseases and schizophrenia, evidence from India is scattered and still in its infancy. Hence, the aims of this paper were to: (1) To comprehensively review the available literature on the interface of cardiovascular health and schizophrenia from India; and (2) To use the information from this scoping review to identify gaps and put forth evidence-informed recommendations to improve management of cardiovascular health among persons with schizophrenia in India.

## Materials and Methods

### Search Strategy

**Figure 1:**
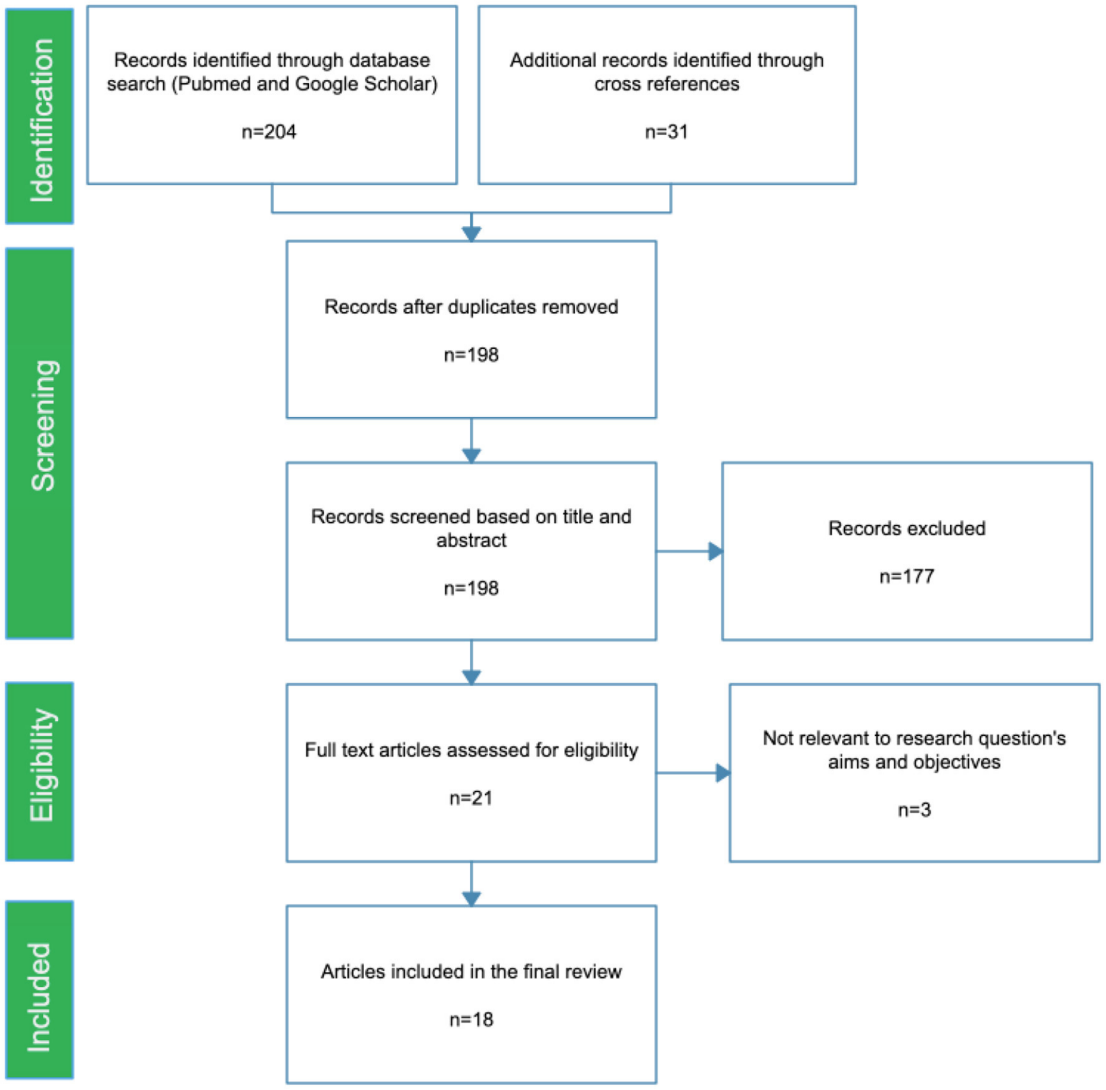
PRISMA flow diagram of the scoping review.

We conducted a scoping review for published articles, up to October 2020. The electronic databases used were PubMed and Google Scholar, using Medical Subject Headings (MESH) terms in combinations: schizophrenia OR psychosis AND “cardiovascular disease” OR “metabolic syndrome” OR diabetes OR hypertension AND India OR Indian, to identify the relevant research publications. Cross references from the articles were retrieved and grey literature were also screened for relevant articles (Figure 1).

### Study Selection

In this scoping review, we included peer reviewed studies, including original research and reviews, that reported on: (1) Prevalence/incidence of cardiovascular disorders in persons with schizophrenia; (2) Etiological and risk factors associated with cardiovascular disorders among persons with schizophrenia; and (3) Intervention/management of cardiovascular disorders among persons with schizophrenia. The inclusion criteria were: (1) Articles on cardiovascular disease and metabolic syndrome—prevalence, risk factors, and interventions for persons with schizophrenia, and studies conducted in India. Commentaries and perspectives were excluded from this review.

### Data Analysis

The research studies obtained from electronic databases were screened according to the inclusion and exclusion criteria. The selected studies were then categorised under relevant sub-headings pertaining to cardiovascular risk.

## Results

### Studies Included

The search Identified 198 unique articles. Of these, 177 articles were excluded as they were not found to satisfy the inclusion and exclusion criteria during the title and abstract search. The remaining 21 research articles were reviewed as full text articles 18 articles were found to be eligible to be included in the current review. All of the studies included in this review, along with their main findings, are given in Table 1.

**Table 1:** Studies on cardiovascular diseases among persons with schizophrenia from India.

**References**	**Study type**	**Variables studied**	**Major findings**
Joshi et al. (5)	Case control	Schizophrenia, cardiovascular disease	•Metabolic syndrome prevalence−28.8% •51.1%higher compared to control group
Saddichha et al. (6)	RCT	Schizophrenia, weight, BMI	•Prevalence of overweight-22.7%; obesity-31.8% •Prevalence of obesity is over 30 times as that of the matched healthy control group
Grover et al. (7)	Cross-sectional	Schizophrenia, neurocognition, metabolic syndrome	•Metabolic syndrome was indicated to have an effect on the neurocognition of those with schizophrenia- cognitive processing and selective attention, auditory and verbal memory, and executive functions
Bijjal et al. (8)	Prospective cohort	Schizophrenia, metabolic syndrome	•Proportion of persons with metabolic syndrome did not increase significantly in this rural cohort
Das et al. (9)	Cross-sectional	Schizophrenia, metabolic syndrome	•78.7% were found to have metabolic abnormalities •Risks identified were female gender, smoking, anti-psychotic use, f/h/o chronic lifestyle disorder
Ganesh et al. (10)	Review	Schizophrenia, metabolic syndrome	•Community based studies highlight a significantly lower prevalence
Rawat et al. (11)	Cross-sectional	Schizophrenia, metabolic syndrome, antipsychotics	•31.8%persons and 28.9% controls were found to have metabolic syndrome •Risk factors were female gender and antipsychotic use
Grover et al. (12)	Cross-sectional	Schizophrenia, metabolic syndrome adolescents	•After starting clozapine the prevalence metabolic syndrome increased from 23 to 38.5% and after 6 months increased to 46.2% •Though other factors prior to onset of medications contribute, Clozapine contributes to half the risk of onset of metabolic changes in adolescence
Anjum and Bathla (13)	Cross-sectional	Schizophrenia, metabolic syndrome	•More than 1/5th psychiatric persons are affected by metabolic syndrome
Rohatgi et al. (14)	Preliminary study	Schizophrenia, metabolic syndrome sleep apnoea, antipsychotic	•Metabolic syndrome in participants taking second-generation antipsychotics is mediated through obstructive sleep apnoea
Kavoor et al. (15)	Case control	Schizophrenia, lipids	•In persons the HDL, LDL levels were found to be lower •Lipid fractions were found to be contributing to levels of impulsivity, suicidality and aggression among persons with schizophrenia
Malhotra et al. (16)	Cross-sectional	Schizophrenia, metabolic syndrome	•Metabolic syndrome was found to be associated with lower scores on health responsibility and nutrition habit, physical activity and stress •Obesity was found to be associated with poor self-esteem and excessive personal distress
Poojari et al. (17)	Retrospective cohort	Schizophrenia, metabolic syndrome antipsychotic	•Age >50 years (OR = 2.00) and duration of antipsychotic treatment>5 years (OR = 1.55) were found to be risk factors •Documenting metabolic changes was inadequate
Anjum et al. (18)	Cross-sectional	Schizophrenia, metabolic syndrome	•Most common metabolic abnormality was low HDL in 76.6%; High TGs in 26.6%; High SBP ≥ 130 mm Hg in 16.67%; DBP>85 mm Hg in 13.33%; High FBS 10% of the persons. In risk assessment strongest risk factors for metabolic syndrome were high waist circumference, FBS, and TGs •In-drug naïve persons, High Density Lipoprotein cholesterol, BMI, and Low Density Lipoprotein were indicated to be risk factors
Gurusamy et al. (19)	Review	Schizophrenia, metabolic syndrome psychoeducation, diet and physical activity interventions	•Non pharmacological management—psychoeducation, diet, and physical activity were proven to be effective in reducing anti-psychotic induced weight gain
Gandhi et al. (20)	Qualitative study	Schizophrenia, metabolic syndrome healthy lifestyle	•Four major themes as facilitators; increased self- confidence, social support and conducive environment; level of self-motivation; encouragement from health professional and availability of health services
Grover et al. (21)	Cross-sectional	Schizophrenia, metabolic syndrome CVD	•Prevalence of metabolic syndrome among healthy controls was 6%, significantly less than persons with SMI •Persons with bipolar disorder had a greater risk for metabolic syndrome
Padmavati et al. (22)	Case control	Schizophrenia, obesity, metabolic syndrome	•Schizophrenia in the absence of antipsychotics was not indicated to contribute to the onset of metabolic syndrome

### Major Findings

Search results indicate that there is a dearth of studies on the interface of CVD and schizophrenia from India, the majority of the studies have examined the prevalence of metabolic syndrome and its association with CVD and the clinical outcomes in persons with schizophrenia.

#### Metabolic Syndrome Among Persons With SMD

Reported literature indicates that standard methods of defining metabolic syndrome have been adopted in the studies, facilitating the comparison and generalizability of the findings. The prevalence of metabolic syndrome among persons with schizophrenia varies in studies from India. In a study conducted in Assam, 78.7% of persons with schizophrenia were found to have metabolic abnormalities (9).There is a higher prevalence amongst persons with mental illness than healthy controls (21). In a recent review by Ganesh et al., pooled prevalence of metabolic syndrome in persons with schizophrenia was 29.83%, and the meta-analysis showed an OR = 3.03 for prevalence in persons with schizophrenia when compared to normal controls and drug-naïve persons had a pooled prevalence of 11.86% (10).

Modifiable lifestyle factors seem to contribute largely by affective quality of life, self-esteem, and increasing distress to the occurrence of metabolic syndrome (16). Some risk factors identified were female gender, antipsychotic use, high BP in men, age > 30 years (13), fasting blood sugar, and triglycerides (18, 23).

Monitoring of metabolic parameters was indicated to be inadequate (17). In drug naïve persons with schizophrenia, high density lipoprotein cholesterol, BMI, and low density lipoprotein were indicated to be risk factors for metabolic syndrome (18).

The presence of metabolic syndrome was also found to impact multiple factors such as neurocognition of persons with schizophrenia (7), while lipid fractions were found to be associated with levels of impulsivity, suicidality, and aggression (15) in persons with schizophrenia. It is further indicated that the presence of mental illness with co-morbid CVD is found to lead to productivity loss (24).

Olanzapine was found to have the greatest weight gain, followed by risperidone and haloperidol (5.1, 4.1, and 2.8 kg), respectively (6). Though other factors contribute to the onset of metabolic changes, clozapine use contributed largely, as after starting clozapine the prevalence of metabolic syndrome increased from 23 to 38.5% and after 6 months increased to 46.2% (12). A study from north India indicates that obstructive sleep apnoea may be a mediating factor for metabolic syndrome with persons on second generation antipsychotics (14). At the same time, Padmavati et al. found low prevalence of obesity and metabolic syndrome among never treated persons with schizophrenia (22). Similarly, the proportion of persons with metabolic syndrome did not increase significantly in this rural cohort, despite the fact that nearly three-fourths of the persons were initiated on second-generation antipsychotics (8).

#### Prevalence of Obesity Among Persons With SMD

The prevalence of overweight individuals was 22.7% and obesity at 31.8%. It was found that at times the difference of prevalence of metabolic syndrome is up to 30 times greater in persons with mental illness when compared to controls (6).

#### Interventions for CVD Among Persons With SMD

Some methods to reduce weight gain due to medications that were proven to be effective were—reducing body mass index, reducing waist circumference, lower blood glucose levels, and interventions by dieticians and nurses (19).Other facilitators to improve healthy lifestyle behaviours were increased self-confidence, social support, and conducive environment; level of self-motivation; encouragement from health professional, and availability of health services (20).

## Discussion

The aims of our study were to synthesise the available information on the interface of schizophrenia and cardiovascular diseases in India through a scoping review of available studies from India, to understand the gaps in the understanding of cardiac diseases in the Indian context and to discuss a potential way forward to improve clinical care and research for CVD among persons with schizophrenia.

Two important risk factors identified have been metabolic syndrome and obesity, both of which could be intervened with. Most of the studies from India have concentrated on the risk factors for CVD such as metabolic syndrome and anti-psychotic medications among persons with schizophrenia (10). There is sparse research on interventions that can prevent the syndrome or manage the components after they manifest (25), despite evidence of effective management using therapeutic lifestyle approaches and targeted pharmacological interventions in the general population (26).

Obesity is a critical factor associated with an increased risk of developing cardiovascular disease. Several meta-analyses have documented the increased prevalence of obesity in persons with mental disorders in general (27).

In India, a large proportion of the population is affected by obesity (28) and the prevalence of obesity in India varies due to various socio-demographic factors. According to an ICMR-INDIAB study in 2015 (29), the prevalence rate of obesity and central obesity varies from 11.8 to 31.3% and 16.9 to 36.3%, respectively. Several meta-analyses have documented the increased prevalence of obesity in persons with mental disorders in general (27) but very few studies from India. This implies the need for more data on obesity, especially central obesity. This becomes important given that there are few intervention studies that cite body weight and patterning as outcome variables in the general population (30) and virtually none in persons with schizophrenia.

Global literature recognises that over three quarters of deaths from heart disease happen in LMIC with 4–5 persons dying of a heart attack or a stroke (31). These outcomes would be avoidable if there was more awareness of the conditions and the ways to control risk factors through lifestyle interventions and drug treatment where necessary (32). In the Indian context too, cardiovascular diseases are the leading cause of mortality, the estimated prevalence being 54.4 million (33). CVDs with ischemic heart disease and stroke responsible for >80% are the cause of one in four deaths (34). These diseases tend to affect persons in the most productive years of their lives and result in catastrophic social and economic consequences.

### Gaps

The gaps in the current knowledge of the status of cardiac health in persons with schizophrenia need to be explored in depth. The association between cardiac disease and mental illnesses has been well-documented with the existing world literature providing relevant insights (35).

The scoping review that we undertook however, demonstrates the limited literature in the Indian context. This finding needs to be understood from several angles. First, it appears that the focus of research has been related largely to metabolic syndrome—taking into context the role of psychotropic medications. Secondly, mental health services are largely delivered in mental health settings, limiting the scope of physical health screening, although the incorporation of psychiatric services at a general hospital psychiatric unit has been documented historically and is within the scope of the National Mental Health program (36). However, despite making mental health services more accessible, several limitations bring about inadequate utilisation of this facility for general medical care for the persons. With increasing emphasis on the primary care provider's role in promoting preventive care, lifestyle changes, and patient self-management, services for the chronically mentally ill persons from the mainstream has become more marginalised (37). Thirdly, there is sufficient evidence that people with schizophrenia are less likely to be screened for lifestyle factors, insufficiently tested for baseline physical parameters or receive standard levels of care for chronic diseases (38). Other factors that limit treatment for cardiac and other physical comorbidities include stigma and diagnostic overshadowing (39). This also limits the possibility of undertaking collaborative research for the study of medical morbidity in persons with severe mental illnesses, explaining the sparse data that is available, despite the increased prevalence of chronic disease and mental illnesses.

Existing literature has several implications. Persons, clinicians and the health care system all play a role in mitigating the multiple factors that are associated with poor physical health in persons with serious mental illnesses. Most persons with schizophrenia receive psychiatric services as out-persons. While many receive medication, social, and rehabilitative services, most either do not receive or access medical care. For persons with serious mental illness, negotiating a separate, complex, medical health care system can be challenging (40). While integrated services offering both mental and medical care at the same location, sometimes even by the same clinician, can overcome systems-based barriers, it also implies that the mental health service system must be able to recognise the comorbidity early and take steps to manage it through referral pathways or collaborative care.

### Recommendations

#### Clinical

Various factors have been identified to contribute to poor cardiovascular health from previous studies. One of the major factors contributing to poor physical health among persons with SMD is lifestyle risk factors (41). Persons with SMD are more likely to smoke, even after comparison with lower socioeconomic status (42). Persons with SMD are also less likely to exercise (43), have diets higher in fat and lower in fibre (44), and are more prone to substance misuse (45). Depressive symptoms (46) and antipsychotic medications (47) have also been implicated to play a role towards poor physical health among persons with SMD. Previous research has highlighted that the mental health professionals are poor in identifying and treating physical health disorders including cardiovascular diseases in persons with SMD (48–50). Improper recording of cardiovascular risk factors and inadequate action are done to intervene to improve these risk factors (49). The stigma of mental illness may be another hurdle that prevents persons from receiving the appropriate and timely treatment (50).

In the recent World Health Organization (WHO) guidelines on managing physical health conditions in persons with severe mental disorders, recommendations are suggested to address important areas such as tobacco cessation, weight management, substance use disorders, cardiovascular disease and cardiovascular risk, diabetes mellitus, HIV/AIDS, and other infectious diseases (tuberculosis, hepatitis B/C) (51).

Physical health screening such as historical review of physical health symptoms and existing chronic disease status and review and follow up of weight, waist circumference, and body mass index are highly recommended to identify CVD risks and diseases early in persons with SMD (52). The recent India Hypertension Control Initiative (IHCI) advocates for regular blood pressure checking for all the persons and caregivers visiting any health facility and initiation of medications at the facility itself rather than referring to experts to prevent delay and drop-out from taking medications (53). Guideline based laboratory assessment of blood glucose, lipid profile, renal functions and ECG monitoring are to be added (51).

As mental health professionals are found to be less involved in the physical health care of persons with SMD (54), additional training to the mental health professionals are needed with respect to advice on safe exercises and diet. At the systems level, efforts must be made to record history of mental illnesses in the data base of the National Hypertension control program (55), to bring in a holistic care for non-communicable disorders in India.

#### Research

Though literature indicates a well-established need for focus on physical health in persons with mental illness, little progress has been made in understanding the implementation barriers in a developing country such as India. The economic disparity in our nation coupled with lack of awareness may contribute largely to multiple comorbid physical problems that may easily go undetected. Due to cultural barriers in India, persons often do not report their concerns freely unless probed by a professional. Due to their difficulty to verbalise their ailments, their pain behaviour, discomfort may often be overshadowed by their psychiatric ailments.

There are a dearth of trained mental health professionals in India, which results in the existing professionals being over burdened with patient load. This may often lead to an oversight while attending to a patient whose physical ailments are not reported specifically by a patient or caregiver. There is a lack of access to medical records across health care professionals which further widens the gap and hinders collaborative care.

Healthy lifestyle behaviours are not common knowledge in rural parts of India. With the easy availability of fast foods and low priced packaged food there has also been an increase in consumption of fatty foods across the nation (56). This coupled with the existing diet in underdeveloped areas which often lack nutritive value may facilitate poor physical health (57). Sedentary lifestyles are perpetuated by a lack of education regarding exercising, lack of access to parks, gymnasiums etc. (58). Non pharmacological interventions to improve motivation and lifestyle behaviours may not suffice unless the model is tailored for rural areas, as implementation barriers are specific and vary widely from region to region (59).

Economic difficulties often dictate the quality of care, time of reaching out for health care (60), urgency of care, and implementation of the health care plan (61). Even when diagnosed an asymptomatic individual with a comorbid SMI often does not implement a regimented care plan due to economic constraints (16). When individuals from underprivileged sectors are faced with economic constraints, there is often a trade-off made between immediate respite from their fatiguing lives and long term investment for health care. Often the latter is neglected till the health concern is of imminent nature. This may also stem from their health beliefs and lack of knowledge of the trajectory of illness. Various challenges and recommendations to improve cardiovascular health and diseases among persons with serious mental disorders is provided in Table 2.

**Table 2:** Challenges and recommendations to improve cardiovascular health and diseases among persons with schizophrenia.

**Challenges**	**Recommendations**
**Patient related**	
Health beliefs	•Effective communication to clarify/change beliefs
Lifestyle factors—Diet and Physical activity	•Assess capability and opportunities for physical activity and healthy diet •Set realistic measurable goals •Lifestyle counselling •Culturally appropriate
Comorbidities—substance use and depression	•Adequate and appropriate treatment for the comorbidities
Weight gain due to antipsychotics	•Change to weight neutral psychotropics •Adjunct Metformin can be considered
Stigma	•Community based interventions •Awareness programmes •Integrating treatment of mental health disorders and NCDs
**Health professionals related**	
Attitudes of health professionals towards mentally ill persons	•Adequate orientation and training on mental health for all health professionals •Continued education programs on regular basis
Lack of Skills to assess and manage risk factors of CVD	•Training to develop skills and provide standardised assessment frameworks •Essential equipment for a physical assessment
**Health system related**	
Lack of integration	•Documentation of NCD related data and maintenance of medical records •Holistic approach for non-communicable disorders
Lack of resources	•Training of primary care health professionals
**Evidence related**	
Paucity of research	•Identification and conduct of locally relevant research questions pertaining to CVD in SMD
Lack of collaborative research	•Development of appropriate interventions involving multiple stakeholders

### Strengths and Limitations

This study, through a comprehensive review on cardiovascular health and diseases among persons with serious mental disorders from India, outlines the knowledge gaps and recommendations based on the existing evidence. At the same time, this study is not without limitations. Most of the studies from India on CVD and schizophrenia are heterogenous and have compared various parameters. Given the limited number of studies providing adequate information on CVD and schizophrenia, risk of bias and high heterogeneity, the review findings are unlikely to be valid among different settings in India. Further, a lack of studies from communities and wide age groups could skew our results.

## Conclusion

The high incidence of cardiovascular diseases among persons with schizophrenia are a major public health concern worldwide and in India. Better understanding of the magnitude of the problem and various biological, psychological, and social factors contributing in the interplay between cardiovascular diseases and schizophrenia is much needed from India to develop cost-effective, scalable, and culturally appropriate interventions to prevent and/or reduce the impact of cardiovascular disease among persons with schizophrenia.

## Author Contributions

RP and VR planned, designed the manuscript, and wrote the first draft of the manuscript. SB did the scoping review of the manuscript and wrote the review section. SK and VR did secondary data analysis. SK and SB commented on the draft and contributed to the subsequent drafts. All authors approved the final manuscript and agreed to submit for publication.

## Conflict of Interest

The authors declare that the research was conducted in the absence of any commercial or financial relationships that could be construed as a potential conflict of interest.
